# Infrared thermography in children: a reliable tool for differential diagnosis of peripheral microvascular dysfunction and Raynaud’s phenomenon?

**DOI:** 10.1186/s12969-019-0371-0

**Published:** 2019-10-16

**Authors:** Giorgia Martini, Michela Cappella, Roberta Culpo, Fabio Vittadello, Monica Sprocati, Francesco Zulian

**Affiliations:** 1 Pediatric Rheumatology Unit, Department of Woman and Child Health, University of Padova, Via Giustiniani 2, 35128 Padova, Italy; 2 Pediatric Unit, Santa Maria Nuova Hospital, Reggio Emilia, Italy; 3 Pediatric Unit, Sant’Anna Hospital, Ferrara, Italy

**Keywords:** Raynaud’s phenomenon, Infrared thermography, Child, Diagnosis, Acrocyanosis

## Abstract

**Background:**

Infrared Thermography (IRT) has been used for over 30 years in the assessment of Raynaud Phenomenon (RP) and other peripheral microvascular dysfunctions in adults but, to date, very little experience is available on its use in children for this purpose. The first aim of the study was to assess reproducibility of thermographic examination after cold exposure by comparing inter-observer agreement in thermal imaging interpretation. The secondary aim was to evaluate whether IRT is reliable to diagnose and differentiate peripheral circulation disturbances in children.

**Methods:**

Children with clinical diagnosis of primary Raynaud’s phenomenon (PRP), secondary RP (SRP), acrocyanosis (AC) and age-matched controls underwent sequential measurements of skin temperature at distal interphalangeal (DIP) and metacarpophalangeal (MCP) joints with IRT at baseline and for 10 min after cold challenge test. Intraclass correlation coefficient (ICC) was calculated for inter-rater reliability in IRT interpretation, then temperature variations at MCP and DIP joints and the distal-dorsal difference (DDD) were analysed.

**Results:**

Fourteen PRP, 16 SRP, 14 AC and 15 controls entered the study. ICC showed excellent agreement (> 0.93) for DIPs and MCPs in 192 measures for each subject. Patients with PRP, SRP and acrocyanosis showed significantly slower recovery at MCPs (*p < 0.05*) and at DIPs (*p < 0.001*) than controls. At baseline, higher temperature at DIPs and lower at MCPs was observed in PRP compared with SRP with significantly lower DDD (*p < 0.001*). Differently from AC, both PRP and SRP showed gain of temperature at DIPs and less at MCPs after cold challenge. PRP but not SRP patients returned to DIPs basal temperature by the end of re-warming time. Analysis of DDD confirmed that controls and PRP, SRP and AC patients significantly differed in fingers recovery pattern (*p < 0.05*).

**Conclusion:**

IRT appears reliable and reproducible in identifying children with peripheral microvascular disturbances. Our results show that IRT examination pointed out that PRP, SRP and AC patients present significant differences in basal extremities temperature and in re-warming pattern after cold challenge therefore IRT can be suggested as an objective tool for diagnosis and monitoring of disease.

## Background

Infrared thermography (IRT) is a diagnostic imaging technique that can record a two-dimensional map of the cutaneous temperature distribution. Since temperature of the skin depends on the local blood perfusion IRT provides important indirect information concerning local circulation. The functional evaluation of vascular reactivity in both basal conditions and in response to different stimuli can be also performed by IRT imaging [1, 2].

Therefore, IRT has been used for over 30 years in the assessment of Raynaud Phenomenon (RP) and other peripheral microvascular dysfunctions in adults but, to date, very little experience is available on its use in children for this purpose [3]. Several thermographic protocols for the assessment of RP comprise a local cold challenge test in attempt to reduce blood flow to mimic the effect of an attack of RP in vivo. The characteristics of the re-warming curve following cold challenge have been successfully applied to differentiate RP patients from healthy controls and IRT has been recently proposed as an objective outcome measure for treatment efficacy trials [4–9].

RP is classified as primary RP (PRP) when it occurs without evidence of an underlying disease and this accounts for approximately 80% of cases, secondary (SRP) when it is associated with other diseases, mainly connective tissue diseases such as systemic sclerosis (SSc), mixed connective tissue disease (MCTD) and systemic lupus erythematosus (SLE) [10]. In children, RP involves about 15% of population with prevalence in females and increasing with age [11, 12]. In an article by Nigrovic et al. the large majority (70%) of RP in children is primary, while the CTD most frequently associated with SRP is Systemic Sclerosis (SSc) where it represents the first sign of the disease in 61–70% of patients [12–14].

Acrocyanosis appears as a symmetric, painless, discoloration of different shades of blue in the distal parts of the body. It is characterized by worsening by cold exposure and frequent association with local hyperhidrosis of hands and feet. The differential diagnosis between acrocyanosis and RP is mainly clinical but sometimes a clear-cut distinction between the two conditions is difficult as some Authors even consider acrocyanosis as a variant of RP [15, 16].

The first aim of the present study was to determine the reproducibility of thermographic examination after cold exposure by comparing inter-observer agreement in thermal imaging interpretation in a paediatric population. The secondary purpose was to evaluate the reliability and diagnostic value of IRT detection of hands temperature before and during re-warming after cold challenge test by comparing children with microvascular dysfunction such as PRP, SRP, acrocyanosis and healthy controls.

## Materials and methods

### Subjects

Patients with age less than 16 years visiting our Paediatric Rheumatology outpatient clinic with personal history of episodes of finger discoloration upon cold exposure and undergoing thermography of their hands entered a cross-sectional study. PRP was defined when episodic reversible bi or-triphasic colour changes in the extremities were not associated with established or suspected connective tissue disease (CTD). SRP was termed when a defined diagnosis of CTD was present at moment of IRT examination. Acrocyanosis was defined when discoloration of extremities was referred as symmetric and persistent. Demographic data, autoantibodies profile and capillaroscopy results were collected. Exclusion criteria were presence of any skin or joint alteration potentially interfering with thermal analysis and any ongoing treatment for RP. The control group consisted of healthy age-matched subjects with absent or mild vascular disturbance on extremities, such as cold fingers after cold exposure but without colour changes. IRB approval was not needed as IRT examination is used in our standard assessment of patients with RP and the present study included only analysis of temperature data. Indeed, written informed consent was obtained from parents of all subjects taking part in the study.

### Thermography measurement protocol and analysis

All subjects were asked not to smoke or consume hot or caffeine-containing beverages as well as take drugs or make physical exercise for at least 4 h prior to the test. Thermographic images were acquired with the same infrared camera (ThermaCAM PM695, FLIR systems AB, Stockholm, Sweden) by same examiner (GM), as previously described [6]. After initial acclimatization of each patient in a temperature-controlled room (at 23 ± 2 °C) for 20 min, thermographic images were taken of the dorsal aspect of both hands (pre-test). The subject then put on latex gloves and placed his/her hands to the metacarpophalangeal (MCP) joints into 15 °C water for 1 min. Gloves were worn for the cold challenge to avoid evaporative cooling and removed immediately for re-warming, then thermographic images of the dorsal aspect of both hands were recorded at one-minute intervals for 10 min during re-warming (time T_0_ to T_10_ of the test).

**Fig. 1:**
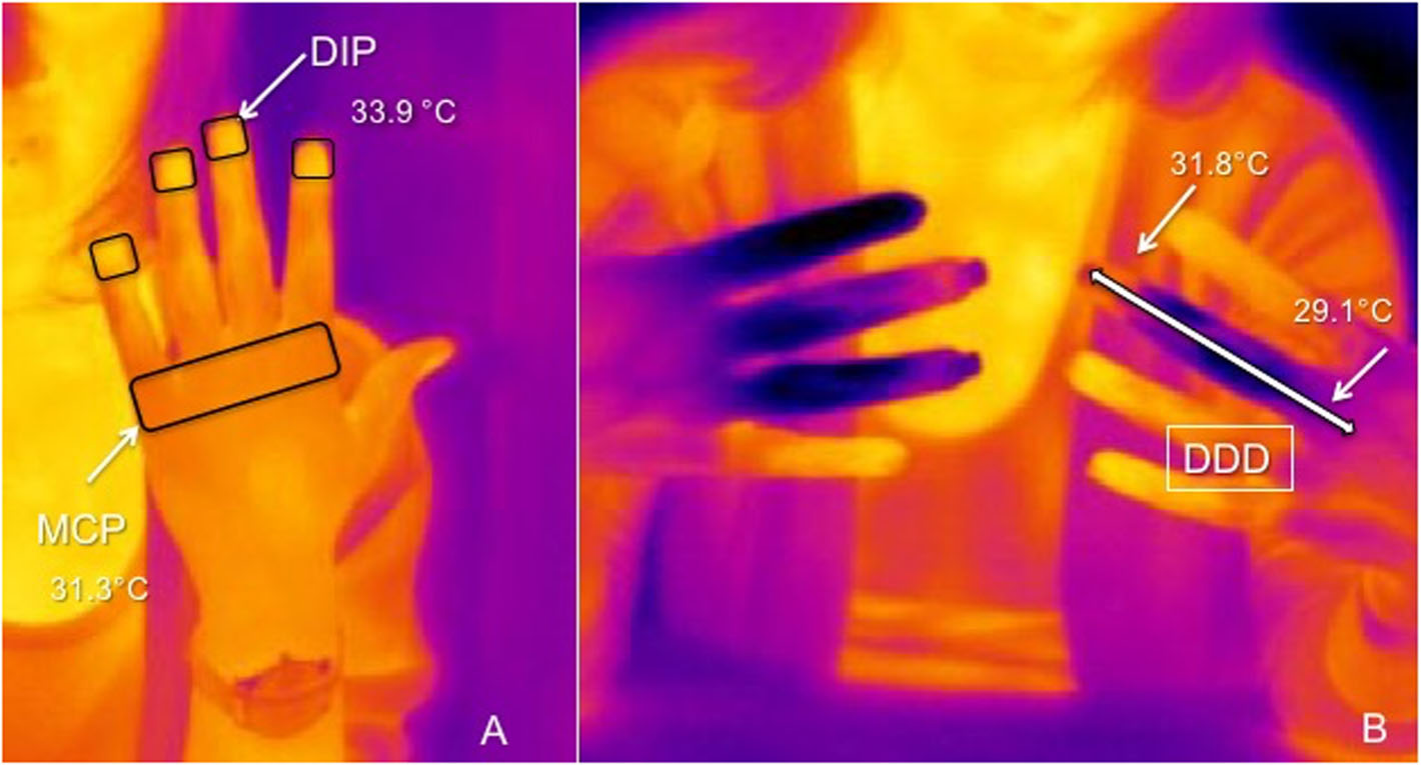
**a** thermographic images showing the areas of temperature measurement at metacarpophalangeal (MCP) and distal interphalangeal (DIP) joints on the dorsal aspect of hands. **b** the measurement of the distal-dorsal difference (DDD) on the III finger of a girl with secondary Raynaud’s phenomenon

Thermographic images were independently and blindly evaluated by two physicians with low or null experience in IRT (MC and RC) and no specific training was performed. For each image acquired before and during the test (pre-test, T_0_ to T_10_) the following data were measured: mean temperature at dorsum of MCP and distal interphalangeal joints (DIP) of the II, III, IV and V finger of both hands as showed in Fig. 1a.

From these measurements the temporal variations of mean temperature at MCP and DIP joints were calculated for each finger in order to evaluate and compare the re-warming patterns. Temporal variation 1 (ΔT_1_) was calculated by deducting temperature at time n from temperature at time n-1 (T_n_-T_n-1_), with n being the minute after cold challenge. Temporal variation 2 (ΔT_2_) was calculated by subtracting the temperature at time n from temperature pre-test (T_n_- T_pre_).

The distal-dorsal difference (DDD) was calculated by subtracting the mean fingertip temperature (DIP) from the mean temperature at dorsum (MCP), as previously described [17, 18]. Therefore, if the fingertip was colder than the dorsum, the DDD was positive (Fig. 1b).

### Statistical analysis

Inter-rater reliability was determined by Intraclass Correlation Coefficient (ICC) and results were interpreted as follows: ICC values range 0.75–1 excellent reliability, 0.4–0.74 good reliability, < 0.4 poor reliability.

The significance of temperature differences between two groups (RP and acrocyanosis vs. controls and Raynaud vs. acrocyanosis) was assessed using the Student’s t-test and Mann-Whitney U test, as appropriate. Temperature variations at MCP and DIP for each finger were evaluated by ANOVA two ways (time and group) for repeated measures. A value of *p* < 0.05 (two-tailed test) was considered to be significant. All statistical analyses were performed by using IBM SPSS (Vers. 18.0).

## Results

### Patient characteristics

Forty-four patients and 15 age-matched controls were included in the study. Sixteen patients were affected by SRP, 14 by PRP and 14 by acrocyanosis (AC). Mean age was 11.9 years (3.6–16) in SRP, 12.2 years (6.1–15.6) in PRP and 14.2 years (6–16.6) in AC patients. Patients with SRP were affected by dSSc (10 patients), lSSc (3), MCTD (2), Overlap syndrome SLE/SSc (1). Mean disease duration was 3.3 years in SRP, 2.2 years in PRP and 1.5 years in AC patients. All SRP and PRP patients were currently reporting RP attacks, while digital ulcers had occurred in 6/16 SRP and in 2/14 PRP patients but none was active at the time of cold challenge. Thirteen patients with SRP were taking treatment (12 calcium channels blockers, 1 ACE inhibitors), 6/14 PRP patients were taking calcium channels blockers.

In SRP patients nail fold capillaroscopy showed scleroderma pattern active in 8 (50%) patients, late in 3 (18.8%), early in 1 (6.3%) and non-specific abnormalities in 4 (25%); in PRP patients showed no abnormalities in 8 (50%) and non-specific findings in 8 (50%), while in AC patients showed acrocyanosis pattern in 8 (mildly reduced capillary density and presence of dilated capillaries). The mean age of healthy controls was 12.4 years of age (8.5–15.8) and 5 of them reported to have “cold hands” but never colour changes. Two patients in the PRP group had low titre (< 1/160) positive antinuclear antibodies but none of them developed any clinical feature of CTD so far. Characteristics of subjects are summarized in Table 1.

**Table 1:** Patients demographics. Data presented as *n* (%) unless stated

	PRP *(n = 14)*	SRP *(n = 16)*	AC *(n = 14)*	Controls *(n = 15)*	*p*
Mean age at assessment *(range)*	12.2 *(6.1–15.6)*	11.9 *(3.6–16)*	14.2 *(6–16.6)*	12.4 *(8.5–15.8)*	*ns*
Gender	10F, 4 M	11F, 5 M	7F, 7 M	12 F, 3 M	*ns*
Underlying diagnosis	–		–	–	
*dSSc*		10 (62.5)			
*lSSc*		2 (12.5)			
*MCTD*		2 (12.5)			
*SLE*		1 (6.3)			
*Overlap (SSc/SLE)*		1 (6.3)			
Antibody profile					
*ANA*	2	16 (100)	1 (8.3)		
*ACA*	–	6 (37.5)	–		
*Topo-1*	–	7 (43.8)	–		
*U1-RNP*	–	6 (37.5)	–		
Capillaroscopy					
Scleroderma pattern active	–	8 (50)	–		
Scleroderma pattern late	–	3 (18.8)	–		
Scleroderma pattern early	–	1 (6.3)	–		
Non-specific	7 (50)	4 (25)	–		
Acrocyanosis pattern	–	–	8 (57.1)		

*Legend: PRP* Primary Raynaud’s phenomenon, *SRP* Secondary Raynaud’s phenomenon, *AC* Acrocyanosis, *dSSc* Diffuse systemic sclerosis, *lSSc* Limited systemic sclerosis, *MCTD* Mixed connective tissue disease, *SLE* Systemic Lupus Erythematosus; Overlap, Overlap syndrome; ns, non-significant, *ACA* anticentromere antibody, *ANA* Antinuclear antibody, *Topo-1* anti-topoisomerasis-1

Each examiner independently and blindly rated a set of 192 measures for each patient and control as II, III, IV and V fingers of both hands were evaluated at MCP and DIP joints at pre-test time and at T_0_ to T_10_ after cold challenge. All IRT examinations were performed in morning hours and without significant differences in seasonal distribution of execution of the procedure between the groups.

### Inter-rater reliability

The inter-rater agreement for temperature measurement at DIP joints was excellent with mean ICC value 0.952 (0.942–0.962) for patients and 0.943 (0.936–0.950) for controls. Similarly, an almost complete agreement between examiners was observed for temperature measurements at MCPs as the mean ICC was 0.955 (0.947–0.964) in the group of patients and 0.945 (0–939-0.951) for controls.

### Analysis of basal temperature

The mean basal temperature at both MCP and DIP joints was significantly lower in patients with PRP, SRP and even more with acrocyanosis compared to controls (*p < 0.001*), as reported in Table 2. Moreover, analysis of DDD showed that, at baseline, patients with PRP presented higher temperature at DIPs and lower at MCPs compared with those with SRP and AC and therefore in PRP the DDD values were significantly higher (*p < 0,001*).

**Table 2:** Mean basal temperature at MCP and DIP joints and DDD in the four groups of subjects

	PRP	SRP	AC	Controls	*p*
Mean DIP temperature Right hand	29.96	29.31	25.66	32.52	*< 0,001*
Mean MCP temperature Right hand	30.51	31.30	28.47	31.93	*< 0,001*
Mean DIP temperature Left hand	29.77	28.82	25.66	32.22	*< 0,001*
Mean MCP temperature Left hand	30.45	31.07	28.29	31.26	*< 0,001*
Mean DDD Right hand *(median)*	0.56 *(0.73)*	1.99 *(1.83)*	2.81 *(2.80)*	−0.59 *(−0.90)*	*< 0,001*
Mean DDD Left hand *(median)*	0.68 *(0.45)*	2.25 *(1.99)*	2.64 *(2.60)*	−0.96 *(− 0.60)*	*< 0,001*

*Legend*: *MCP* Metacarpal-phalangeal joints, *DIP* Distal interphalangeal joints, *DDD* distal-dorsal difference, *PRP* Primary Raynaud’s phenomenon, *SRP* Secondary Raynaud’s phenomenon, *AC* Acrocyanosis

### Analysis of re-warming pattern

**Fig. 2:**
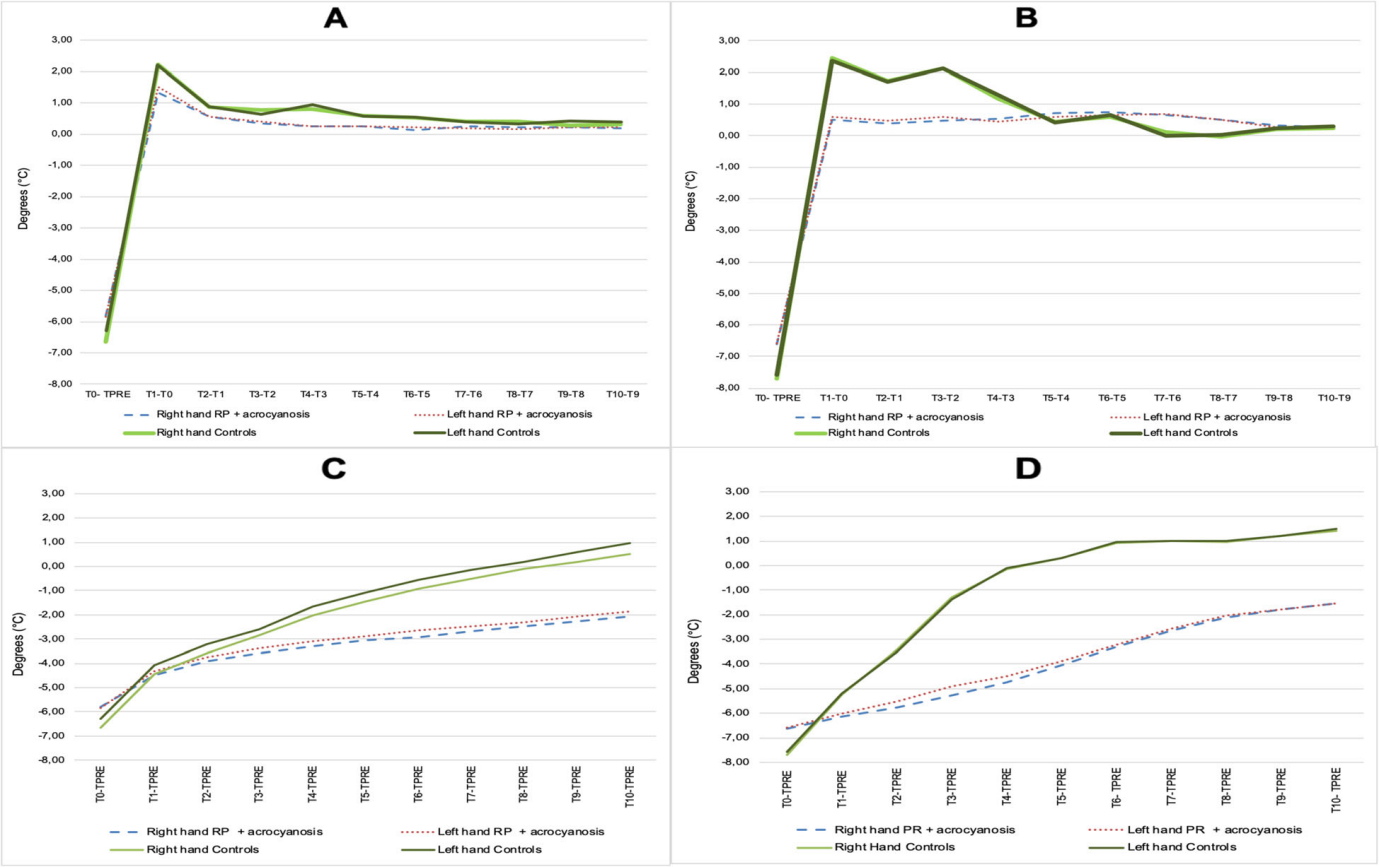
analysis of temperature temporal variations showing the different re-warming pattern in patients (PRP and SRP and acrocyanosis taken together) from controls. In ΔT_1_ controls presented gain of basal temperature significantly earlier at MCPs (**a**) but even more at DIPs (*p < 0.05*), as shown in (**b**). In ΔT_2_ healthy controls reached higher temperatures at MCPs more rapidly than patients (*p < 0.001*) as showed in (**c**), and this difference was even more evident at DIPs (**d**)

The analysis of temperature temporal variations showed that IRT was able to clearly differentiate patients (PRP and SRP and acrocyanosis considered together) from controls. In fact, the re-warming pattern was significantly slower in patients’ group as showed by analysis of ΔT_1_ in which controls presented gain of basal temperature significantly earlier at MCPs, but even more at DIPs (*p < 0.05*) (Fig. 2a and b). This different trend was more evident in the comparison of ΔT_2_, with healthy controls reaching higher temperatures and more rapidly than patients both in MCPs and DIPs (*p < 0.001*) as showed in Fig. 2c and d, respectively.

**Fig. 3:**
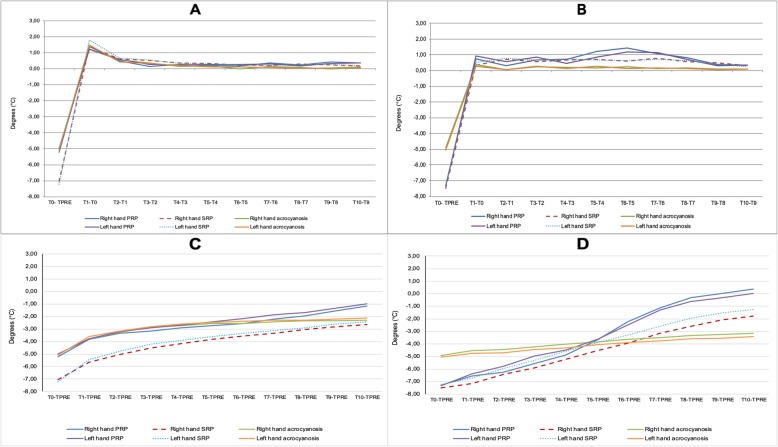
analysis of temperature temporal variations showing the different re-warming pattern in PRP and SRP patients from those with acrocyanosis. In ΔT_1_ analysis subjects with acrocyanosis presented a slower and smaller gain of temperature over time at MCPs and more at DIPs (**a** and **b** respectively). The analysis of ΔT_2_ showed that at MCPs patients with PRP and SRP presented similar re-warming pattern (**c**) with PRP patients reaching higher temperature levels. At DIPs in the 10 min after cold challenge patients with PRP showed to return to basal temperature, differently from SRP and even more from acrocyanosis patients (**d**)

The analysis of re-warming pattern showed that patients with PRP and SRP significantly differed from AC particularly looking at ΔT_2_ temporal variation. Indeed, subjects with both PRP and SRP presented some gain of temperature over time particularly at DIPs and this allowed PRP, but not SRP patients, to achieve the basal temperature by the end of the re-warming time. Inversely, in patients with AC the fingertips temperature after cold challenge showed only null or minimal changes over time. (Fig. 3a-d).

**Fig. 4:**
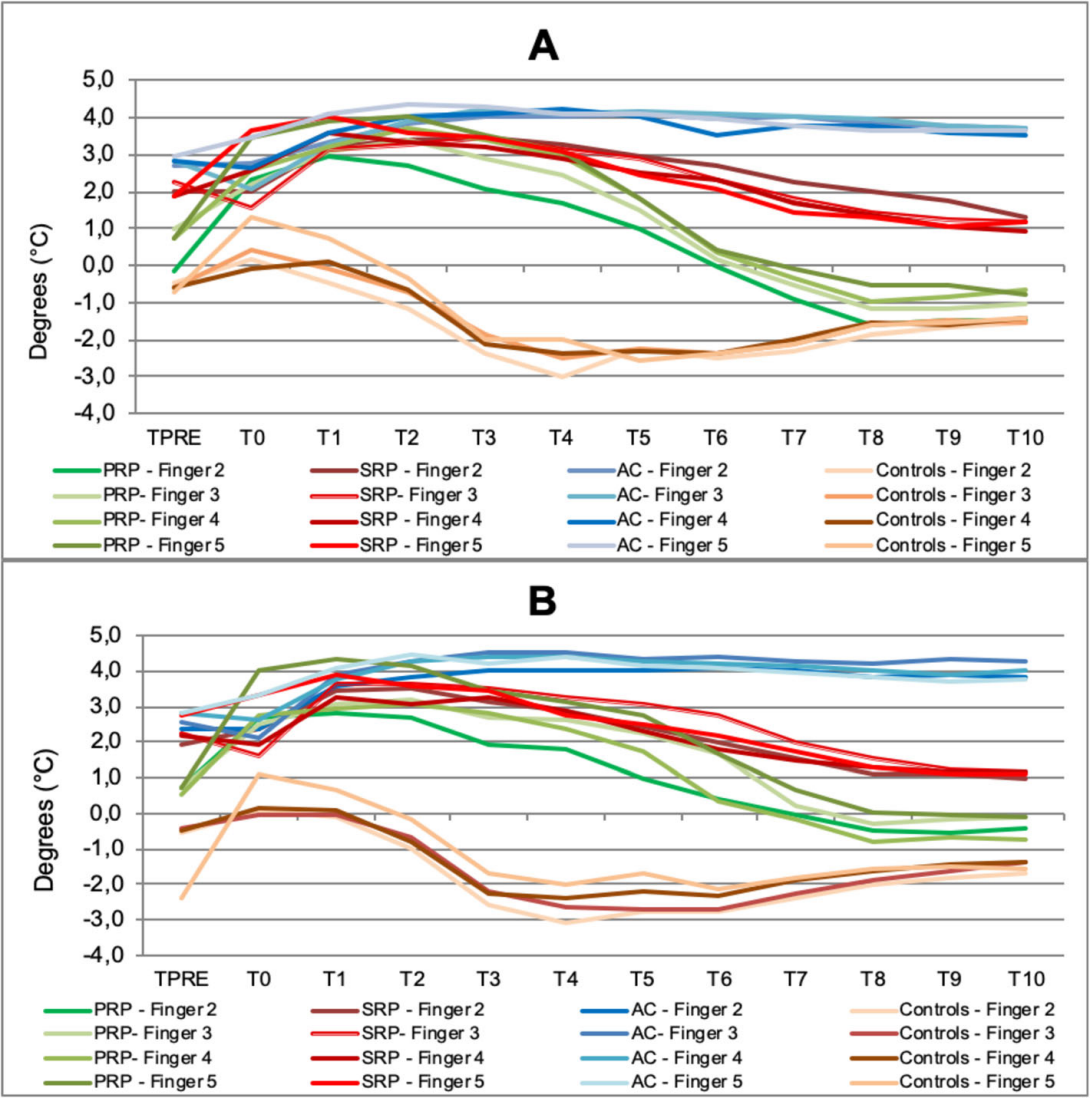
analysis of distal-dorsal difference (DDD) showing the different recovery pattern in controls and in subjects with PRP, SRP and acrocyanosis. PRP patients present smaller DDD at baseline compared to SRP and acrocyanosis; furthermore, during the final part of test a gradual reduction of DDD is observed in both PRP and SRP patients. In particular, in PRP subjects the temperature of DIPs reaches higher levels than basal after 10 min from cold challenge and DDD becomes negative. In acrocyanosis DDD did not show any change during the whole re-warming period (**a** right hand, **b** left hand) (*p < 0.05*)

Furthermore, the analyses of DDD confirmed that patients with PRP, SRP and acrocyanosis significantly differed from healthy controls as for fingers temperature recovery pattern, as showed in Fig. 4a and b. After cold challenge, in PRP patients the fingertips were initially colder than dorsum but difference progressively reduced during the re-warming phase, achieving higher values than basal, as in fact DDD values became negative after 10 min. In SRP patients, the DIPs temperature, after a transitory reduction, gradually increased and DDD got back to the baseline values at the end of the test. Conversely, in acrocyanosis the DIPs temperature increased very slowly over the whole re-warming period (*p < 0.05*).

## Discussion

Infrared thermography (IRT) is an easy to apply and well-established imaging method which showed good reproducibility in healthy subjects and in several pathological conditions such as malignancies, muscular-skeletal inflammation and complex regional pain syndrome (CRPS) [19–21].

Skin vessels dilate or constrict in response to changes of either environmental or internal body temperature and of psycho-physiological state, thus cutaneous micro-circulation is a major effector of thermoregulation. This feature, particularly evident at extremities, is non-specific and individual so the range of hands temperature is very wide among healthy people [22]. IRT has been largely used in adults to assess the peripheral circulation; some protocols in standardized conditions and including cold challenge test have been proposed to diagnose RP and differentiate patients with primary and secondary forms [1, 4–6, 23, 24]. Moreover, a recent large multicentre study clearly demonstrated that IRT is an objective and reliable outcome measure to be used in clinical trials for evaluation of treatments effectiveness [9].

In the paediatric population IRT has been successfully used in diagnosis and monitoring of some pathological conditions like injuries, in order to reduce exposure to ionizing radiations, haemangiomas, vascular malformations, burns, deep venous thrombosis and localized scleroderma [25–27].

Herein we demonstrated that IRT represents a promising and reliable tool for diagnosing and monitoring peripheral circulation disturbance in paediatric patients. The first important result of our study is that the interpretation of thermal images by different physicians with poor experience in IRT was almost completely concordant as ICC value was higher than 0.93 in all measures in patients and controls.

Indeed, IRT provides objective and reproducible measures of blood flow and our data showed that it can help in the identification of patients with definite peripheral microvascular disturbances from healthy children. In fact, although diagnosis of RP and AC is generally clinical, in children a sharp distinction between these conditions and variant of physiological “freezing fingers” can be very difficult solely on the basis of patient’s medical history evaluation. To the best of our knowledge, this is the first demonstration that healthy children, RP and acrocyanosis patients exhibit different thermal dynamic responses to a standard challenge test. In fact, in the recovery time following the test, healthy subjects present a rapid reactive hyperaemia, starting from the fingertips, that often completes before the end of observation, while RP and acrocyanosis patients show a slow protracted recovery of more than 10 min from baseline.

Another interesting point of the present study is the capability of thermal images evaluation to distinguish PRP from SRP and acrocyanosis on the basis of the re-warming pattern analysis. Patients with PRP present a more rapid and greater gain of temperature over time, particularly at DIPs, compared with those with SRP and acrocyanosis. Moreover, the analysis of the longitudinal gradient shows that, after cold challenge, in RP patients the recovery occurs from the distal part of finger while in acrocyanosis the difference between fingertips and MCPs remains stable over time.

These observations can be explained by the different origin of these microcirculation abnormalities. In PRP vascular reactivity is maintained and this allows a rapid recovery, while in SRP the microvasculature is partially compromise, as proved by the abnormal capillaroscopy and by the altered composition of the vasal layers. In acrocyanosis the different behaviour may be explained by the preminent pathogenetic involvement of venous portion of circulation, with reduced venous tone and sub-capillary venous plexus dilatation [16]. Another difference was that in RP the re-warming pattern differed from finger to finger, while in acrocyanosis it was more homogeneous.

Several studies in adults reported that IRT examination is helpful for differentiating PRP from SRP, such as in systemic sclerosis [1, 6, 17, 28–31]. For the first time in paediatric age we showed that, in basal conditions, patients with PRP exhibit higher temperature at DIPs, and subsequent lower DDD values, than those with SRP. Furthermore, during re-warming phase, temperature at DIPs returned to basal values in PRP but not in SRP patients, thus indicating more severely disturbed peripheral circulation.

In previous studies in adults, IRT examination was repeated in consecutive days in order to account potential circadian and seasonal variations, thus one possible limitation of the present study is that cold challenge was performed only once in each patient [9].

The correct identification of patients with definite peripheral microvascular disturbances is prominent in order to define which ones deserve to be further investigated with a diagnostic work-up including auto-antibodies profile and nail fold capillaroscopy. Capillaroscopy is currently one of the most informative techniques for the diagnosis of RP and has been recommended in adults and in children because presence of specific abnormalities in nail fold capillaries is associated with a higher risk of development of a connective tissue disease, such as SSc and SLE [13, 14, 32–35]. In adults capillaroscopy showed high sensitivity and specificity in diagnosis of scleroderma-spectrum disorders, nevertheless it is still an operator-dependent technique so, in order to overcome the potential heterogeneity of images interpretation, continuous EUSTAR/EULAR effort is done to standardize the modality of assessment [36, 37].

In children, capillaroscopy appears feasible and non-invasive but with high possibility of poor-quality images for several factors such as the need of collaboration to keep the hand steady or periungueal region damaging for nail biting, nail/finger traumas or infections. Another limitation is that, in growing healthy children, the microvascular network changes gradually into mature adult form and non-specific microvascular abnormalities, such as capillary tortuosity, can be observed. Recent publications indicated normal patterns in healthy children and adolescents in order to standardize capillaroscopy thus, an in-depth knowledge of the developmental stages and longstanding experience are crucial for the correct interpretation of capillaroscopy images in paediatric age [38–40].

Cold-challenge IRT has the advantage of assessing the microvascular function in a dynamic way that reproduces what happens in the real life. Technical and cost limitations of first-generation infrared cameras restricted the use of IRT in medicine until recently, with improvement in camera technology, costs and data handling. In fact, the small size and weight of modern cameras are similar to domestic camcorders. More recently, a mobile phone thermography came on the market, potentially offering a more affordable and portable alternative to “standard” thermography and showing comparable measurements, therefore easily exploitable in an outpatient setting [9, 24].

IRT examination procedure finds an excellent acceptance by children and their parents and, in our study, the collaboration in the cold challenge test was very good. Indeed, the vision of sophisticated colour images of their hands was felt by school-age and older children as a game and an award for their collaboration in the test.

## Conclusions

Our results, although with the limits of a small population, suggest that IRT appears as a reliable and reproducible method to evaluate children with abnormal peripheral circulation, particularly in cases without a clear-cut clinical picture or presentation. In fact, in our study PRP, SRP and AC patients presented significant differences both at basal observation and during the re-warming phase. These results suggest that IRT can help clinicians to avoid complex diagnostic algorithms in RP-mimicking conditions. Moreover, as recently confirmed in adults, IRT can be suggested as an objective outcome measure to quantify the disease severity and to assess its evolution over time and in response to treatments.

## Data Availability

Dataset generated and analysed during the current study are available from the corresponding author on reasonable request.

## Abbreviations

AC: Acrocyanosis
CRPS: Complex regional pain syndrome
CTD: Connective tissue disorders
DDD: Distal-dorsal difference
DIP: Distal interphalangeal joint
EULAR: European League Against Rheumatism
EUSTAR: European Scleroderma Trials and Research
ICC: Intraclass correlation coefficient (ICC)
IRT: Infrared thermography
MCP: Metacarpophalangeal joints
MCTD: Mixed connective tissue disease
PRP: Primary Raynaud’s phenomenon
RP: Raynaud’s phenomenon
SLE: Systemic lupus erythematosus
SRP: Secondary Raynaud’s phenomenon
SSc: Systemic sclerosis
